# Supplementary material for: A Global Screen for Assembly State Changes of the Mitotic Proteome by SEC-SWATH-MS
Source: Cell Syst. 2020 Feb 26;10(2):133–155.e6. doi: 10.1016/j.cels.2020.01.001 (PMC7042714; doi:10.1016/j.cels.2020.01.001)

Q6UWE0 | LRSM1_HUMAN | LRSAM1 TAL UNQ6496/PRO21356
Monomer MW [kDa]: 83.594 Monomer expected elution fraction: 42

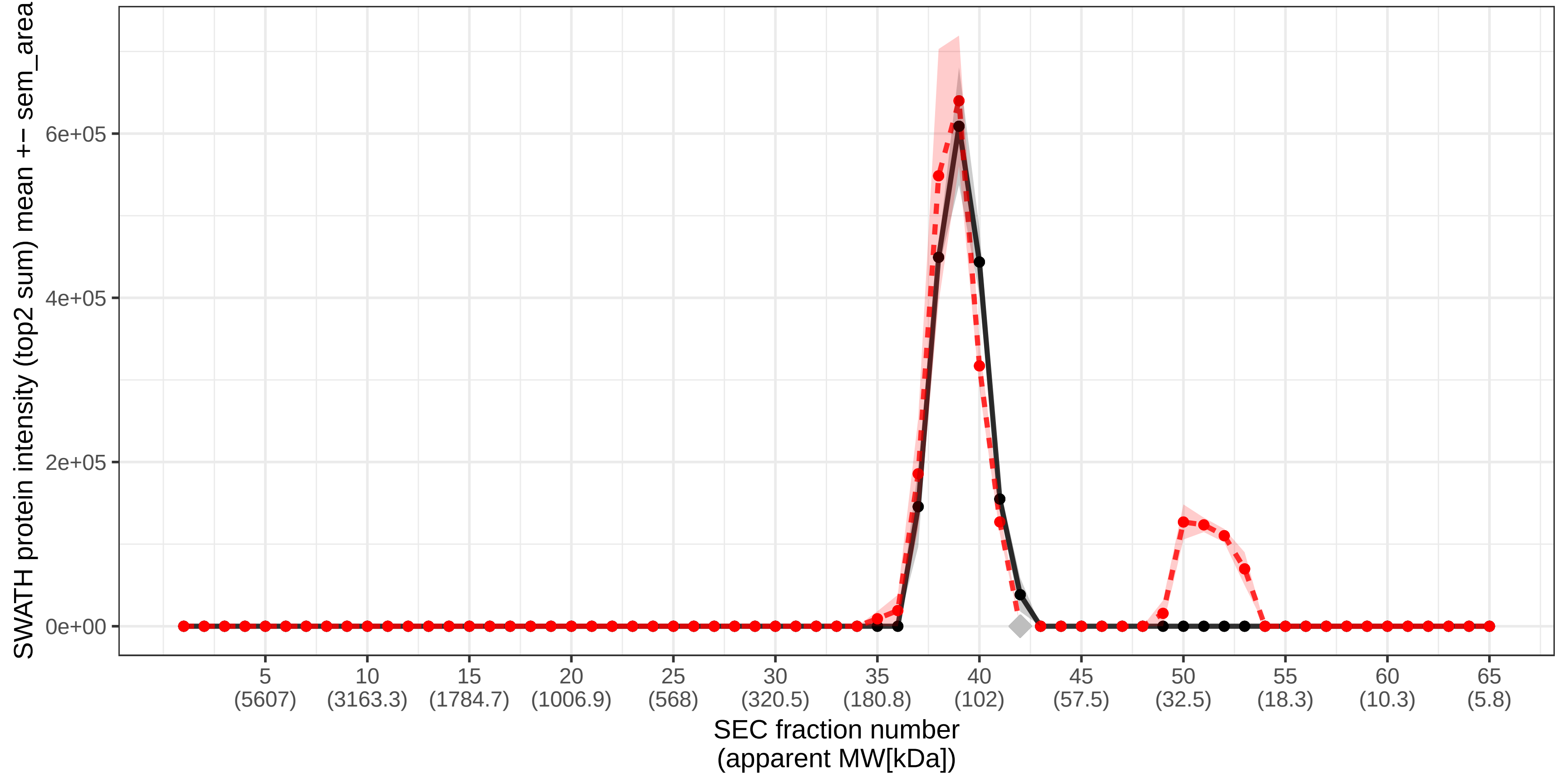

Supplement: Data S1. SEC-SWATH-MS Protein Chromatograms, Related to Figure 1 [file mmc6.zip › SECchrom_Q6UWE0_LRSM1_HUMAN_LRSAM1_TAL_UNQ6496_PRO21356.pdf]
